# In-vitro activity of newly-developed β-lactamase inhibitors avibactam, relebactam and vaborbactam in combination with anti-pseudomonal β-lactam antibiotics against AmpC-overproducing clinical *Pseudomonas aeruginosa* isolates

**DOI:** 10.1007/s10096-024-04965-x

**Published:** 2024-11-26

**Authors:** Christophe Le Terrier, Otávio Hallal Ferreira Raro, Alaaeldin Mohamed Saad, Patrice Nordmann, Laurent Poirel

**Affiliations:** 1 Emerging Antibiotic Resistance Unit, Medical and Molecular Microbiology Unit, Department of Medicine, Faculty of Science, University of Fribourg, Chemin du Musée 18, Fribourg, CH-1700 Switzerland; 2 Division of Intensive Care Unit, University Hospitals of Geneva, Geneva, Switzerland; 3 Swiss National Reference Center for Emerging Antibiotic Resistance, Fribourg, Switzerland; 4 Department of Zoonoses, Faculty of Veterinary Medicine, Zagazig University, Zagazig, Egypt

**Keywords:** β-lactamases inhibitors, Antimicrobial resistance, Vaborbactam, Relebactam, Avibactam, *Pseudomonas aeruginosa*, AmpC overproducing, Cephalosporins, Ceftolozane, Carbapenem

## Abstract

**Purpose:**

Overproduction of the intrinsic chromosomally-encoded AmpC β-lactamase is one of the main mechanisms responsible for broad-spectrum β-lactam resistance in *Pseudomonas aeruginosa*. Our study aimed to evaluate the in-vitro activity of anti-pseudomonal β-lactam molecules associated with the recently-developed and commercially-available β-lactamase inhibitors, namely avibactam, relebactam and vaborbactam, against *P. aeruginosa* isolates overproducing their AmpC.

**Methods:**

MIC values of ceftazidime, cefepime, meropenem, imipenem and ceftolozane with or without β-lactam inhibitor were determined for 50 AmpC-overproducing *P. aeruginosa* clinical isolates. MIC breakpoints for resistance were retained at 8 mg/L for β-lactams and β-lactam/β-lactamase inhibitor combinations containing ceftazidime, cefepime and meropenem, while 4 mg/L was used for those containing imipenem and ceftolozane. The concentration of all β-lactamases inhibitors was fixed at 4 mg/L, except for vaborbactam (8 mg/L).

**Results:**

The rates of isolates not being resistant to ceftazidime, cefepime, meropenem, imipenem and ceftolozane were found at 12%, 22%, 34%, 8% and 74%, respectively. When combined with avibactam, those rates increased to 60%, 62%, 60%, 46%, and 80%, respectively. The highest rates were found with relebactam-based combinations, being 76%, 64%, 66%, 76% and 84%, respectively. By contrast, associations with vaborbactam did not lead to significantly increased “non-resistance” rates.

**Conclusion:**

Our results showed that all combinations including relebactam led to higher “non-resistance” rates against AmpC-overproducing *P. aeruginosa* clinical isolates. The best activity was achieved by combining ceftolozane and relebactam, that might therefore be considered as an excellent clinical alternative against AmpC overproducers.

**Supplementary Information:**

The online version contains supplementary material available at 10.1007/s10096-024-04965-x.

## Introduction

*Pseudomonas aeruginosa* is one of the main pathogens associated with nosocomial infections [1–3]. Indeed, *P. aeruginosa* frequently infects vulnerable patients, the latter having been subject to multiple therapies [4]. The growing threat of antimicrobial resistance in *P. aeruginosa* relies on the capacity of this organism to develop resistance to any available antibiotic linked to different mechanisms [5, 6]. Besides the acquisition of broad-spectrum β-lactamases such as extended-spectrum β-lactamases (ESBLs) or carbapenemases, the main mechanisms responsible for broad-spectrum β-lactam resistance in that bacterial species corresponds to the overproduction of its naturally AmpC-type chromosomally-encoded cephalosporinase (also called *Pseudomonas*-derived cephalosporinases or PDC) resulting in resistance to most anti-pseudomonal penicillins (piperacillin, ticarcillin) and cephalosporins (ceftazidime, cefepime), monobactams such as aztreonam, and even to reduced susceptibility to carbapenems [7, 8]. Based on previous studies, the overall prevalence rate of AmpC-overproducing *P. aeruginosa* was estimated around 20% in bloodstream isolates of *P. aeruginosa*, representing a major concern in clinical practice since the occurrence of multidrug- or even pandrug resistance is frequently observed consequently to antibiotic selective pressure [9–12]. Therefore, novel anti-pseudomonal β-lactams, such as ceftolozane, and novel β-lactam inhibitors (BLIs) have been developed in order to counteract this major health issue [12, 13]. Hence, the ceftolozane-tazobactam combination is particularly interesting against *P. aeruginosa*, nevertheless resistance to this combination is frequently reported and considerably varies between countries from 4 to 45% [11, 12, 14].

Avibactam, relebactam and vaborbactam are newly-developed non-β-lactam BLIs with in-vitro activity against β-lactamases of Ambler class A including extended-spectrum β-lactamases (ESBLs) and KPC-type carbapenemases, some class C β-lactamases (naturally-occurring AmpC enzymes of *P. aeruginosa* or Enterobacterales), and class D enzymes for avibactam (e.g. OXA-48-type) [15–18]. These novel BLIs have been associated with anti-pseudomonal β-lactams, such as ceftazidime-avibactam, imipenem-relebactam, and meropenem-vaborbactam, and are now currently available for clinical use. They may therefore be considered as potential antimicrobial therapies against MDR/XDR *P. aeruginosa* infections given the in-vitro activities ranging from approximately 65–80% [11, 12]. However, the abovementioned β-lactams possess variable antibacterial activities against *P. aeruginosa* strains, and the abovementioned BLI also exhibit variable spectrum and level of inhibitory activities against the various chromosomally-encoded cephalosporinases from *P. aeruginosa*, namely PDC-like β-lactamases. The efficacy of most of these anti-pseudomonal β-lactams is particularly affected when PDC-like enzymes are overproduced and may consequently lead to treatment failure [19]. Therefore, our aim was to evaluate which of the antipseudomonal β-lactam/BLI combination might exhibit the best in vitro activity against *P. aeruginosa* AmpC overproducers.

## Materials and methods

### Screening for non-extended-spectrum β-lactamase (ESBL)- and non-carbapenemase-producing *P. aeruginosa* isolates

From a large set of multidrug-resistant *P. aeruginosa* isolates collected at the Swiss National Reference Center for Emerging Antibiotic Resistance (NARA) between 2018 and 2022 and recovered from patients across all Switzerland, a selection was made on the phenotypic non-susceptibility observed for ceftazidime, defined as MIC value being ≥ 8 mg/L. Then, screening for carbapenemase production was performed before inclusion in the study by using the RAPIDEC^®^ Carba NP test (bioMérieux, Marcy-l’Étoile, France) [20, 21]. On the other hand, producers of ESBLs were screened by searching for the corresponding genes, namely *bla*_VEB−like_, *bla*_GES−like_, *bla*_BEL−1_, and *bla*_PER−1_, using PCR amplification with primers listed in Table S1. Finally, all ESBL- and carbapenemase-producing *P. aeruginosa* were excluded from this study in order to only focus on the problem of AmpC overproduction on its own.

### Selection of isolates compatible with *ampC* overexpression phenotype

For all carbapenemase-negative isolates, an antibiogram was systematically performed with solid Mueller-Hinton (MH) plates supplemented with cloxacillin 2 g/L (supposed to inhibit the impact of the AmpC enzyme on resistance to broad-spectrum cephalosporins) to screen for all phenotypic patterns compatible with *ampC* gene overexpression. The phenotype that was considered for selection of putative AmpC overproducers was defined as follow; a ≤ 17 mm diameter around the ceftazidime disk on MH plates and an increased diameter of at least 5 mm on MH supplemented with cloxacillin at 2 g/L for the same ceftazidime disk, according to EUCAST guidelines [7, 22]. Subsequently, for sake of accuracy in the categorization of AmpC overproducers, the expression level of the respective *ampC* genes was determined by RT-qPCR experiments, as detailed below.

### Expression of *ampC* gene by RT-qPCR

The total RNA of each tested isolate was extracted using the Quick-RNA™ MiniPrep kit (Zymo Research, Irvine, CA, USA) after incubation at 37° C with shaking until reaching growth absorbance (OD at 600 nm) at 0.5–0.6. Then, to remove contaminating DNA from RNA preparations, and DNase and divalent cations from the samples, a turbo DNA-free™ kit (Invitrogen, Waltham, MA, USA) was used. cDNA synthesis was performed with the LunaScript^®^ RT SuperMix kit (New England BioLabs, Ipswich, MA, USA). All experiments were performed following the manufacturer’s instructions. Finally, cDNA samples were measured to assure purity and standard concentration quantification using the NanoDrop 2000 spectrophotometer (Thermo Scientific, Reinach, Switzerland). Quantitative real-time PCR was done using the Rotor-Gene Q cycler (Qiagen, Hilden, Germany). Housekeeping gene *nadB* was used as a control of expression [23]. The primers used in this experiment were those already described for *bla*_ampC_ and *nadB* gene (L-aspartate oxydase) [23, 24]. Reactions were established with a total volume of 20 µL using a GoTaq^®^ qPCR Master Mix kit (Promega, Fitchburg, WI, USA). The cycle threshold (*CT*) values were analyzed by the 2−∆∆CT method [25]. Relative expression levels were calculated by comparing the results with results obtained with *P. aeruginosa* ATCC 27853, and the condition values were corrected with the reference gene. The overproduction of *bla*_ampC_ was considered significant when the corresponding ratio was > 2. Results of the *ampC* gene expression are detailed in Table S2 and S3.

### Susceptibility testing

The MICs values were determined in triplicate by broth microdilution method according to EUCAST guidelines [26]. MICs of all β-lactams including ceftazidime, cefepime, imipenem, meropenem and ceftolozane were determined alone or in combination with a fixed concentration of avibactam (4 mg/L), relebactam (4 mg/L), or vaborbactam (8 mg/L) [17, 27]. Ceftazidime, cefepime and ceftolozane were purchased from Sigma-Aldrich (Saint-Louis, USA), and imipenem, meropenem from HuiChem (Shanghai, China). All the inhibitors (avibactam HY-14879, relebactam HY-16752, vaborbactam HY-19930, tazobactam HY-B1418) were purchased from MedChem Express (Luzern, Switzerland). The *P. aeruginosa* ATCC 27853 and *Klebsiella pneumoniae* ATCC BAA-2814 reference strains were used as quality control for β-lactams and combinations with BLIs, respectively, for all testing [27]. As most susceptible EUCAST breakpoints are ≤ 0.001 mg/L, interpretation was based on EUCAST resistance breakpoints for the β-lactam alone for *P. aeruginosa* [22]. Hence, resistances to ceftazidime and to ceftazidime-BLI combinations were defined as > 8 mg/L, resistances to cefepime and cefepime-BLI as > 8 mg/L, resistances to imipenem and imipenem-BLI as > 4 mg/L, resistances to meropenem and meropenem-BLI as > 8 mg/L, and resistance ceftolozane and ceftolozane-BLI as > 4 mg/L. In addition, the “non-resistance” is defined when MIC value is found lower than EUCAST resistance breakpoints for the antibiotics and combinations aforementioned.

### β-lactamase activities

The *bla*_PDC− 1_ and *bla*_PDC− 5_ genes, respectively encoding PDC-1 and PDC-5 (that latter variant being defined as an extended-spectrum cephalosporinase [ESAC]) [7], were cloned into the shuttle vector pUCP24, and expressed in *E. coli* TOP10 recipient strains. To evaluate the respective inhibitory activities of each BLI against PDC-like enzymes, cultures of *E. coli* TOP10 harbouring recombinant plasmids respectively producing PDC-1 and PDC-5 (ESAC) were grown overnight at 37 °C in 50 ml of Luria broth with gentamicin (50 mg/L). The bacterial suspension was pelleted, resuspended in 10 mL of 100 mM phosphate buffer (pH 7), sonicated using a Vibra cell™ 75,186 sonicator (Thermo Fisher Scientific) and centrifuged for 1 h at 11,000 x g and 4 °C. The β-lactamase activity was monitored using nitrocefin (200 µM). Kinetic measurements were performed at room temperature in 100 mM sodium phosphate (pH 7.0) using a UV/visible ULTROSPEC 2100 pro spectrophotometer (Amersham Biosciences, Buckinghamshire, UK). Cephalothin wavelength and absorption coefficient were used (262 nm/Ʌ ɛ = -7960 M -1 cm − 1) [29]. Fifty-percent inhibitory concentrations (IC_50_) were determined for clavulanic acid, tazobactam, avibactam, relebactam, and vaborbactam. Various concentrations of these BLIs were pre-incubated with the crude extract of the enzyme for 3 min at room temperature to determine the concentrations that reduced the hydrolysis rate of 100 µM cephalothin by 50%. Results were expressed in micromolar units.

## Results

### Antimicrobial susceptibility to the different anti-pseudomonal β-lactam/BLI combinations

After phenotypic and genotypic screening as detailed in the Material and Methods section, a collection of 50 AmpC-overproducing *P. aeruginosa* clinical isolates was retained in the study. Among all these AmpC-overproducing *P. aeruginosa* clinical isolates, 12% were categorized as non-resistant to ceftazidime, exhibiting MIC values at 8 mg/L, being the exact EUCAST breakpoint, as shown in Table 1. The non-resistant rates for cefepime, meropenem, imipenem and ceftolozane were 22%, 34%, 8% and 74%, respectively. For the different β-lactam/BLI combinations tested, that actually correspond to those already available on the market, ceftolozane-tazobactam was the most effective one, followed by imipenem-relebactam with non-resistant rates at 80% and 76%, respectively, considering MIC ≤ 4 mg/L. Considering the actual EUCAST breakpoint for imipenem-relebactam (2 mg/L) the non-resistant rate for this latter combination was 60%. The highest non-resistant rates were found with relebactam-based combinations, namely ceftazidime-relebactam (76% MIC ≤ 8 mg/L), cefepime-relebactam (64% MIC ≤ 8 mg/L), meropenem-relebactam (66% MIC ≤ 8 mg/L), imipenem-relebactam (76% MIC ≤ 4 mg/L; 60% MIC ≤ 2 mg/L) and ceftolozane-relebactam (84% MIC ≤ 4 mg/L), respectively. When combined with avibactam, those rates slightly decreased to 60%, 62%, 60%, 46%, and 80%, respectively. Interestingly, associations with vaborbactam did not display significantly increased non-resistant rates compared to the β-lactam alone, being at 14%, 28%, 38%, 8%, and 72%, respectively, suggesting a poor inhibitory activity of vaborbactam against PDC-like enzymes. Detailed MIC values results of all *P. aeruginosa* clinical isolates overproducing their AmpC are shown in Table S2.

**Table 1:** Cumulative MIC distribution of ceftazidime, cefepime, imipenem, meropenem and ceftolozane in the presence and absence of avibactam, relebactam, vaborbactam and tazobactam in 50 AmpC overexpression of *Pseudomonas aeruginosa* strains

ß-lactam	ß-lactamase inhibitor in combination	Cumulative % of isolates at MIC (mg/L)												% of isolates with MIC values (mg/L)	
		≤ 0.125	0.25	0.5	1	2	4	8	16	32	64	≥ 128	≤ 8		≥ 16
Ceftazidime	alone							**12**	26	46	62	100	12		88
	+ Avibactam 4 mg/L					8	50	**60**	72	78	84	100	60		40
	+ Relebactam 4 mg/L				2	26	50	76	84	90	92	100	76		24
	+ Vaborbactam 8 mg/L						4	14	26	48	64	100	14		86
Cefepime	alone					2	6	**22**	50	66	84	100	22		78
	+ Avibactam 4 mg/L					14	36	62	72	88	94	100	62		38
	+ Relebactam 4 mg/L				2	16	44	64	74	86	94	100	64		36
	+ Vaborbactam 8 mg/L						10	26	54	74	88	100	28		72
Meropenem	alone			2	6	8	14	**34**	70	80	88	100	34		66
	+ Avibactam 4 mg/L	2	4	8	12	14	44	60	82	92	96	100	60		40
	+ Relebactam 4 mg/L		4	8	14	18	48	66	90	94	94	100	66		34
	+ Vaborbactam 8 mg/L		2	6	8	10	18	**38**	62	80	90	100	38		62
													≤ 4		≥ 16
Imipenem	alone				2	4	**8**	10	46	80	94	100	8		90
	+ Avibactam 4 mg/L	2	4	6	14	28	46	84	94	98	98	100	46		16
	+ Relebactam 4 mg/L	2	4	4	28	**60**	76	92	94	96	100	100	76		8
	+ Vaborbactam 8 mg/L				2	4	8	14	56	78	98	100	8		86
Ceftolozane	alone			4	34	60	74	80	86	94	94	100	74		20
	+ Tazobactam 4 mg/L			10	32	62	**80**	86	90	94	98	100	80		14
	+ Avibactam 4 mg/L		2	26	58	72	80	90	96	96	98	100	80		10
	+ Relebactam 4 mg/L		4	46	68	80	84	90	98	100	100	100	84		10
	+ Vaborbactam 8 mg/L		2	4	40	58	72	78	88	94	96	100	72		22

In bold, the cumulative % of isolates at the actual resistant EUCAST breakpoint v. 14.0 (2024)

Among the isolates tested, 11 and 8 exhibited MIC values ≥ 8 mg/L for ceftolozane-tazobactam and ceftolozane-relebactam, respectively. Interestingly, although isolates PA36, PA14 and PA11 showed resistance to ceftolozane-tazobactam, MIC values of the ceftolozane-relebactam combination remained < 8 mg/L for those isolates, for which the respective *ampC* gene overexpression levels were found to be 321 x, 18 x and 185 x in comparison with that of the ATCC 27853 reference wild-type strain, as detailed in Table S2. This suggested a better inhibitory effect of relebactam than tazobactam against those PDC variants.

Among the tested collection, a total of 20 isolates were found to be resistant to ceftazidime-avibactam. On the other hand, a total of 12 isolates showed MIC values at > 8 mg/L when combining ceftazidime with relebactam. Finally, nine isolates were resistant to both combinations, as shown in Table S2.

### Fifty-percent inhibitory concentrations for β-lactamase inhibitors against PDC enzymes

In order to better evaluate the respective activities of the different BLIs, namely the “old” clavulanic acid and tazobactam in comparison with the “novel” avibactam, relebactam, and vaborbactam against the chromosomally-encoded AmpC β-lactamase of *P. aeruginosa*, two PDC representative enzymes were used, namely the PDC-1 reference variant and the PDC-5 ESAC variant [7]. Determination of the IC_50_ values was performed using crude enzyme extracts from *E. coli* recombinant strains producing those variants. As expected, clavulanic acid did not show any effective inhibitory activity against PDC-like enzyme. On the other hand, avibactam and relebactam showed similar moderate activities even though they were the best inhibitors of the PDC-like enzymes. Noteworthy, PDC-1 and PDC-5 were poorly inhibited by vaborbactam as suggested by the high IC_50_ value observed for this BLI, in line with the antimicrobial susceptibility results using vaborbactam as BLI (Table 2).

**Table 2:** Specific ß-lactamase activities and inhibitory concentrations for ß-lactamase inhibitors

Enzyme	IC_50_ (µM)				
	Clavulanic acid	Tazobactam	Avibactam	Relebactam	Vaborbactam
PDC-1	> 100	14.9	1.4	3	11.3
PDC-5	> 100	18.3	0.9	1.9	36.7

## Discussion

In this study, we showed that all BL/BLI combinations including relebactam as BLI had higher in-vitro activity compared to the other BLIs tested, against AmpC-overproducing *P. aeruginosa*. In addition, our data showed that ceftolozane-relebactam, a newly-evaluated combination that does actually not correspond to a marketed one, achieved the optimal antibacterial activity, including against AmpC-overproducing and carbapenem-resistant isolates.

IC_50_ data suggest that the inhibitory activity of relebactam against PDC-type enzymes is similar to avibactam, mirroring results from previous reports [18, 19, 30]. Indeed, it was previously reported that relebactam possesses an excellent capacity to inhibit the activity of AmpC enzymes, particularly the intrinsic PDC-like β-lactamases of *P. aeruginosa* [31]. Then, it is important to highlight that relebactam may be less affected by inoculum effect and production of efflux pumps, in comparison to avibactam, regardless of the underlying PDC allele [31, 32]. Also, avibactam likely induces the *ampC* expression of *P. aeruginosa*, by contrast to relebactam [24]. A previous study reported a significant and interesting activity of the imipenem-relebactam combination against imipenem-resistant *P. aeruginosa* isolates, supporting that relebactam is less affected by combination of OprD deficiency, AmpC overproduction and efflux, the most prevalent resistance mechanisms identified in that species [33–35].

Of note, the ceftolozane-tazobactam combination has been previously shown to exhibit great efficiency against AmpC-overproducing *P. aeruginosa*, mainly because ceftolozane is not (or weakly) hydrolyzed by most PDC enzymes, and this antibiotic also possesses a stronger affinity than ceftazidime and imipenem for penicillin-binding-proteins (PBPs) of *P. aeruginosa*. In addition, ceftolozane was shown to be a very weak inducer of the *ampC* gene expression [36–38]. Our results showing a high in-vitro effectiveness (74%) of ceftolozane alone are in line with previous studies [34, 39]. Combined with relebactam, only eight isolates showed MICs values above the ceftolozane breakpoint. Nevertheless, even if the antibacterial activity of ceftolozane is weakly affected by the hydrolysis activity of PDC enzymes, certain specific PDC variants demonstrate a greater affinity for ceftolozane, resulting in increased hydrolysis and resistance to ceftolozane-tazobactam [19, 40]. Conversely, relebactam, a more efficient inhibitor of PDC than tazobactam, when associated with ceftolozane, can therefore prevent hydrolysis of a wider spectrum of PDC variants.

Our data showed high rates of ceftazidime-avibactam and imipenem-relebactam resistance among the isolates tested, with both resistance rates of 40%, considering 2 mg/L as breakpoint for imipenem-relebactam according to EUCAST guidelines. This might be explained by the overproduction of PDC-like enzymes, but might also be related to other resistance mechanisms such as efflux or OprD deficiency, features that have not been evaluated in the present study. In a large collection of ceftazidime-resistant *P. aeruginosa*, Stone GG. et al. found a susceptibility rate of 63.8% for ceftazidime-avibactam, which correlates with our results [41].

Our study also highlighted the lack of significant benefit when combining β-lactams with vaborbactam as BLI, compared to the β-lactam alone. The poor in-vitro activity of meropenem-vaborbactam compared to meropenem alone against AmpC overproducers of *P. aeruginosa* was recently reported [42–44]. Our data reinforce these observations, with determination of IC_50_ values confirming the poor inhibitory activity of vaborbactam toward PDC-like enzymes, as previously described [18].

To conclude, this study highlights the in-vitro effective activity of using relebactam-based combinations with antipseudomonal β-lactams, the most effective combination corresponding to ceftolozane-relebactam against such AmpC-overproducing *P. aeruginosa* isolates. This combination might therefore be considered as a promising alternative. While we acknowledge that the combination ceftolozane-relebactam is not currently available on the market, we believe that our findings may encourage the susceptibility testing of ceftolozane-tazobactam and imipenem-relebactam together and the use of both combinations in association when dealing with isolates resistant to the commercialized ceftolozane-tazobactam, ceftazidime-avibactam and imipenem-relebactam combinations.

## Electronic supplementary material

Below is the link to the electronic supplementary material.

Supplementary Material 1

## Data Availability

No datasets were generated or analysed during the current study.
