# Supplementary material for: In-vitro activity of newly-developed β-lactamase inhibitors avibactam, relebactam and vaborbactam in combination with anti-pseudomonal β-lactam antibiotics against AmpC-overproducing clinical Pseudomonas aeruginosa isolates
Source: Eur J Clin Microbiol Infect Dis. 2024 Nov 26;44(2):277–84. doi: 10.1007/s10096-024-04965-x (PMC11754317; doi:10.1007/s10096-024-04965-x)
Supplement: Supplementary file 1 — Supplementary Material 1 [file 10096_2024_4965_MOESM1_ESM.docx]

| **ESBL gene** | **Direction** | **Primer sequence** | **Amplicon size** |
| --- | --- | --- | --- |
| *bla*_VEB_ | Forward | 5’-CGA CTT CCA TTT CCC GAT GC-3’ | 643 nt |
|  | Reverse | 5’-GGA CTC TGC AAC AAA TAC GC-3’ |  |
| *bla*_GES_ | Forward | 5'-ATG CGC TTC ATT CAC GCA C-3' | 864 nt |
|  | Reverse | 5'-CTA TTT GTC CGT GCT CAG G-3' |  |
| *bla*_BEL_ | Forward | 5’-GAA AGG TTC GCG ATG TGC TC-3' | 493 nt |
|  | Reverse | 5’-CAG GCA AGC CAG CTC GAA TG-3’ |  |
| *bla*_PER_ | Forward | 5’-ATG AAT GTC ATT ATA AAA GC-3’ | 925 nt |
|  | Reverse | 5’-AAT TTG GGC TTA GGG CAG AA-3’ |  |

**Table S1.** Primers used for PCR amplification and detection of extended-spectrum ß-lactamase-producing *P. aeruginosa* isolates

|  | Clinical isolate | PDC-like variant | AmpC expression 2^-ΔΔCT^ | Minimal Inhibitory Concentration (mg/L)^a^ | | | | | | | | | | | | | | | | | | | | |
| --- | --- | --- | --- | --- | --- | --- | --- | --- | --- | --- | --- | --- | --- | --- | --- | --- | --- | --- | --- | --- | --- | --- | --- | --- |
|  |  |  |  | **Ceftazidime** | | | | **Cefepime** | | | | **Imipenem** | | | | **Meropenem** | | | | **Ceftolozane** | | | | |
|  |  |  |  | alone | AVI | REL | VAB | alone | AVI | REL | VAB | alone | AVI | REL | VAB | alone | AVI | REL | VAB | alone | TAZ | AVI | REL | VAB |
|  | ATCC  27853^b^ | PDC-1 | 1 | 0.5 | 0.5 | 0.5 | 0.5 | 0.5 | 0.5 | 0.5 | 0.5 | 0.5 | 0.5 | 0.5 | 0.5 | 0.25 | 0.25 | 0.25 | 0.25 | 0.25 | 0.25 | 0.25 | 0.25 | 0.25 |
|  | ATCC 2814^c^ | - |  | 2,048 | 8 | 8 | 16 | 512 | 0.5 | 1 | 2 | 32 | ≤0.12 | ≤0.12 | 0.125 | 128 | ≤0.12 | ≤0.12 | 0.25 | 128 | 128 | 1 | 2 | 4 |
| Imipenem non-resistant PA isolates | PA8 | PDC-1 | 27 | 64 | 16 | 2 | 64 | 16 | 2 | 2 | 16 | 1 | 0.125 | 0.125 | 1 | 1 | 0.5 | 0.5 | 0.5 | 1 | 1 | 0.5 | 0.25 | 2 |
|  | PA16 | PDC-15 | 28 | 64 | 8 | 2 | 32 | 16 | 4 | 4 | 8 | 2 | 0.25 | 0.25 | 2 | 1 | 0.25 | 0.25 | 0.5 | 1 | 1 | 0.5 | 0.5 | 1 |
|  | PA19 | PDC-109 | 77 | 64 | 16 | 8 | 64 | 16 | 8 | 4 | 16 | 4 | 0.5 | 1 | 4 | 8 | 4 | 4 | 8 | 2 | 2 | 1 | 0.5 | 4 |
|  | PA20 | PDC-3 | 59 | 256 | 128 | 8 | 128 | 128 | 4 | 4 | 128 | 4 | 1 | 1 | 4 | 8 | 2 | 1 | 1 | 1 | 1 | 1 | 0.5 | 1 |
| Imipenem-resistant  PA isolates | PA1 | PDC-16 | 16 | 8 | 4 | 2 | 4 | 8 | 8 | 4 | 8 | 64 | 16 | 2 | 64 | 16 | 8 | 8 | 16 | 1 | 0.5 | 0.5 | 0.5 | 1 |
|  | PA2 | PDC-63 | 2.4 | 16 | 4 | 4 | 16 | 8 | 4 | 4 | 8 | 16 | 8 | 4 | 16 | 16 | 16 | 16 | 8 | 1 | 0.5 | 1 | 0.5 | 1 |
|  | PA3 | PDC-501 | 2.3 | 256 | 16 | 8 | 64 | 32 | 32 | 32 | 32 | 32 | 8 | 8 | 32 | 16 | 8 | 8 | 16 | 4 | 4 | 4 | 4 | 4 |
|  | PA4 | PDC-15 | 595 | 64 | 32 | 8 | 64 | 128 | 8 | 4 | 64 | 64 | 8 | 2 | 64 | 64 | 32 | 16 | 64 | 4 | 4 | 1 | 0.5 | 4 |
|  | PA5 | PDC-501 | 148 | 128 | 4 | 8 | 128 | 32 | 32 | 16 | 32 | 16 | 4 | 2 | 16 | 16 | 4 | 4 | 8 | 4 | 4 | 2 | 2 | 2 |
|  | PA6 | PDC-4 | 10 | 16 | 2 | 2 | 16 | 8 | 8 | 8 | 8 | 16 | 4 | 4 | 16 | 8 | 4 | 4 | 8 | 1 | 1 | 1 | 1 | 1 |
|  | PA7 | PDC-3 | 6 | >256 | 4 | 8 | 128 | 64 | 32 | 8 | 64 | 16 | 1 | 1 | 16 | 64 | 16 | 16 | 64 | 1 | 1 | 1 | 0.5 | 1 |
|  | PA9 | PDC-36 | 20 | 16 | 4 | 2 | 32 | 8 | 2 | 2 | 8 | 16 | 1 | 2 | 16 | 8 | 4 | 4 | 8 | 1 | 1 | 1 | 1 | 2 |
|  | PA10 | PDC-1 | 186 | 256 | 64 | 128 | 128 | 128 | 128 | 128 | 128 | 64 | 16 | 8 | 32 | 128 | 128 | 128 | 64 | 8 | 8 | 8 | 8 | 16 |
|  | PA11 | PDC-1 | 185 | 256 | 128 | 32 | >128 | 256 | 16 | 8 | 64 | 64 | 16 | 16 | 64 | 64 | 16 | 16 | 128 | 8 | 8 | 8 | 1 | 8 |
|  | PA12 | PDC-433 | 19 | 128 | 128 | 4 | 128 | 64 | 64 | 128 | 32 | 32 | 8 | 8 | 32 | 64 | 8 | 4 | 128 | 2 | 2 | 1 | 0.5 | 64 |
|  | PA13 | PDC-3 | 13 | 16 | 8 | 4 | 128 | 2 | 4 | 1 | 4 | 32 | 32 | 8 | 64 | 256 | 4 | 4 | 32 | 128 | 128 | 128 | 32 | 128 |
|  | PA14 | PDC-5 | 18 | 256 | 128 | 32 | >128 | 256 | 128 | 128 | 128 | 32 | 32 | 64 | 64 | 256 | 64 | 128 | 128 | 128 | 4 | 4 | 1 | 8 |
|  | PA15 | PDC-7 | 5.8 | 16 | 4 | 2 | 8 | 32 | 16 | 16 | 16 | 128 | 16 | 2 | 64 | 16 | 16 | 16 | 16 | 4 | 4 | 4 | 2 | 4 |
|  | PA17 | PDC-5 | 426 | 128 | 128 | 8 | 128 | 32 | 16 | 16 | 32 | 16 | 8 | 2 | 16 | 32 | 64 | 32 | 32 | 8 | 4 | 1 | 1 | 8 |
|  | PA18 | PDC-51 | 13 | >256 | 4 | 8 | >128 | 64 | 8 | 8 | 64 | 16 | 2 | 1 | 16 | 8 | 1 | 0.5 | 4 | 32 | 2 | 0.5 | 2 | 32 |
|  | PA21 | PDC-12 | 37 | 128 | 4 | 32 | 128 | 64 | 2 | 16 | 128 | 32 | 8 | 4 | 8 | 4 | 4 | 2 | 4 | 2 | 2 | 0.5 | 0.5 | 1 |
|  | PA22 | PDC-470 | 17 | 8 | 16 | 8 | 8 | 8 | 8 | 8 | 8 | 32 | 8 | 4 | 16 | 16 | 16 | 16 | 32 | 0.5 | 1 | 0.5 | 0.5 | 0.5 |
|  | PA23 | PDC-196 | 4.5 | 256 | 64 | 128 | 128 | 64 | 32 | 32 | 32 | 16 | 8 | 4 | 16 | 16 | 16 | 8 | 16 | 16 | 16 | 16 | 8 | 16 |
|  | PA24 | PDC-3 | 2.8 | 8 | 4 | 8 | 8 | 4 | 4 | 4 | 4 | 32 | 16 | 32 | 32 | 8 | 4 | 4 | 8 | 1 | 0.5 | 1 | 0.5 | 1 |
|  | PA25 | PDC-36 | 33 | 16 | 4 | 4 | 16 | 128 | 128 | 64 | 128 | 16 | 8 | 8 | 16 | 256 | 128 | 128 | 128 | 32 | 64 | 64 | 16 | 32 |
|  | PA26 | PDC-36 | 13 | 32 | 32 | 8 | 32 | 8 | 8 | 8 | 8 | 16 | 2 | 8 | 64 | 4 | 4 | 4 | 4 | 1 | 1 | 1 | 0.5 | 1 |
|  | PA27 | PDC-3 | 72 | 128 | 128 | 128 | 128 | 128 | 64 | 64 | 64 | 16 | 4 | 2 | 16 | 4 | 4 | 4 | 4 | 16 | 8 | 8 | 8 | 16 |
|  | PA28 | PDC-53 | 10 | 16 | 2 | 16 | 16 | 16 | 2 | 4 | 8 | 128 | 4 | 4 | 16 | 128 | 4 | 4 | 128 | 2 | 1 | 0.5 | 0.5 | 1 |
|  | PA29 | PDC-60 | 14 | 32 | 4 | 2 | 32 | 16 | 2 | 4 | 16 | 16 | 8 | 1 | 16 | 32 | 4 | 4 | 32 | 2 | 2 | 0.25 | 0.25 | 2 |
|  | PA30 | PDC-5 | 4 | 32 | 16 | 16 | 32 | 32 | 32 | 16 | 32 | 16 | 4 | 2 | 8 | 32 | 32 | 16 | 64 | 1 | 1 | 1 | 1 | 1 |
|  | PA31 | PDC-36 | 8 | 32 | 4 | 2 | 16 | 64 | 16 | 32 | 16 | 32 | 8 | 2 | 16 | 128 | 16 | 8 | 16 | 4 | 4 | 2 | 2 | 4 |
|  | PA32 | PDC-3 | 25 | 64 | 8 | 8 | 64 | 16 | 8 | 8 | 16 | 16 | 8 | 2 | 16 | 16 | 32 | 16 | 16 | 2 | 2 | 1 | 1 | 2 |
|  | PA33 | PDC-36 | 4.6 | 8 | 8 | 8 | 8 | 64 | 64 | 64 | 64 | 32 | 8 | 8 | 32 | 8 | 8 | 8 | 8 | 2 | 4 | 4 | 2 | 4 |
|  | PA34 | PDC-3 | 6 | 8 | 2 | 1 | 4 | 8 | 2 | 2 | 4 | 32 | 2 | 1 | 16 | 8 | 1 | 1 | 16 | 1 | 0.5 | 0.5 | 0.5 | 0.25 |
|  | PA35 | PDC-35 | 35 | 128 | 64 | 16 | 128 | 64 | 32 | 32 | 32 | 16 | 8 | 2 | 16 | 16 | 16 | 16 | 32 | 2 | 2 | 2 | 0.5 | 2 |
|  | PA36 | PDC-35 | 322 | 128 | 32 | 4 | 128 | 32 | 8 | 8 | 32 | 16 | 8 | 4 | 16 | 16 | 16 | 8 | 32 | 128 | 64 | 8 | 2 | 128 |
|  | PA37 | PDC-3 | 16 | 128 | 4 | 2 | 64 | 16 | 4 | 2 | 16 | 32 | 4 | 2 | 32 | 16 | 4 | 4 | 32 | 2 | 2 | 1 | 0.5 | 1 |
|  | PA38 | PDC-3 | 43 | 32 | 2 | 4 | 32 | 16 | 2 | 2 | 16 | 64 | 8 | 1 | 32 | 16 | 4 | 4 | 8 | 1 | 2 | 2 | 0.5 | 1 |
|  | PA39 | PDC-5 | 13 | 64 | 4 | 2 | 64 | 16 | 4 | 2 | 16 | 32 | 4 | 1 | 32 | 8 | 8 | 8 | 16 | 2 | 2 | 0.5 | 0.5 | 1 |
|  | PA40 | PDC-5 | 25 | 64 | 4 | 2 | 64 | 16 | 4 | 2 | 16 | 32 | 4 | 1 | 32 | 8 | 8 | 4 | 8 | 2 | 2 | 0.5 | 0.5 | 2 |
|  | PA41 | PDC-3 | 21 | 32 | 4 | 4 | 32 | 16 | 8 | 4 | 16 | 32 | 2 | 2 | 32 | 16 | 8 | 4 | 32 | 0.5 | 0.5 | 0.5 | 0.5 | 1 |
|  | PA42 | PDC-3 | 14 | 256 | 128 | 64 | 128 | 32 | 32 | 32 | 64 | 16 | 8 | 4 | 16 | 16 | 16 | 16 | 16 | 32 | 32 | 16 | 16 | 32 |
|  | PA43 | PDC-1 | 15 | 64 | 8 | 4 | 32 | 16 | 8 | 4 | 16 | 64 | 4 | 2 | 64 | 16 | 16 | 16 | 16 | 4 | 4 | 2 | 4 | 4 |
|  | PA44 | PDC-501 | 10 | >256 | 4 | 16 | >128 | 256 | 32 | 64 | 128 | 16 | 2 | 2 | 16 | 2 | 0.5 | 1 | 2 | 16 | 16 | 16 | 16 | 16 |
|  | PA45 | PDC-3 | 4.6 | 16 | 16 | 4 | 16 | 8 | 8 | 8 | 8 | 8 | 4 | 1 | 8 | 32 | 32 | 16 | 32 | 1 | 1 | 0.5 | 0.5 | 1 |
|  | PA46 | PDC-501 | 20 | 8 | 4 | 4 | 8 | 4 | 4 | 4 | 4 | 16 | 2 | 1 | 16 | 0.5 | 0.125 | 0.25 | 0.25 | 1 | 2 | 1 | 1 | 1 |
|  | PA47 | PDC-5 | 79 | 32 | 2 | 4 | 32 | 8 | 4 | 4 | 4 | 64 | 8 | 8 | 64 | 16 | 4 | 8 | 8 | 2 | 2 | 1 | 1 | 2 |
|  | PA48 | PDC-3 | 3.2 | 32 | 4 | 2 | 16 | 16 | 8 | 8 | 16 | 32 | 8 | 2 | 64 | 8 | 4 | 2 | 8 | 4 | 2 | 2 | 1 | 1 |
|  | PA49 | PDC-23 | 2.1 | 256 | 128 | 128 | 128 | 64 | 16 | 32 | 32 | 128 | 128 | 64 | 128 | 32 | 32 | 32 | 64 | 32 | 32 | 8 | 16 | 16 |
|  | PA50 | PDC-3 | 7.1 | 32 | 4 | 2 | 32 | 16 | 8 | 8 | 16 | 32 | 2 | 4 | 32 | 16 | 8 | 8 | 16 | 2 | 2 | 2 | 1 | 2 |

^a^ß-lactamase inhibitor abbreviations: AVI, avibactam at 4 mg/L; REL, relebactam at 4 mg/L ; VAB, vaborbactam at 8 mg/L; TAZ, tazobactam at 4 mg/L. ^b^QC strain *P. aeruginosa* ATCC 27853 with inducible AmpC. ^c^QC strain *K. pneumoniae* ATCC BAA-2814 produce KPC-3, SHV-11 and TEM-1.

**Table S2.** MIC distribution of ceftazidime, cefepime, imipenem, meropenem and ceftolozane in the presence and absence of avibactam, relebactam, vaborbactam and tazobactam in 50 AmpC overexpression of *Pseudomonas aeruginosa* strains.

| **Detailed statistics of AmpC expression 2^-ΔΔCT^** | **Number of fold compared to results with *P. aeruginosa* ATCC 27853** |
| --- | --- |
| Mean | 54.7 |
| Standard Error | 15.8 |
| Median | 16.1 |
| Interv Interq 25% | 6.8 |
| Interv Interq 75% | 35.6 |
| Standard Deviation | 111.9 |
| Sample Variance | 12515.3 |
| Kurtosis | 13.0 |
| Skewness | 3.5 |
| Range | 592.2 |
| Minimum | 2.1 |
| Maximum | 594.3 |
| Sum | 2734.9 |
| Count | 50 |
| Largest | 594.3 |
| Smallest | 2.1 |
| Confidence Level (95,0%) | 31.8 |

**Table S3.** Detailed results of *ampC* gene expression of the collection of 50 *Pseudomonas aeruginosa* clinical isolates AmpC overproducers.
